# To be or not to be: how does the brain respond to different infectious agents?

**DOI:** 10.1128/msphere.00326-25

**Published:** 2025-08-05

**Authors:** Ushma Ruparel, Christopher J. Tonkin

**Affiliations:** 1 The Walter and Eliza Hall Institute of Medical Research 5388, Melbourne, Victoria, Australia; 2 Department of Medical Biology, The University of Melbourne 2281, Melbourne, Victoria, Australia; Virginia-Maryland College of Veterinary Medicine, Blacksburg, Virginia, USA

**Keywords:** *Toxoplasma*, immunity, neuron, brain, infection, flavivirus

## Abstract

*Toxoplasma gondii* is an apicomplexan parasite that chronically infects approximately 30% of the global population. In healthy adults, infection is typically resolved within a few weeks, resulting in tissue cysts that persist in the central nervous system for the lifetime of the host. In immune-compromised patients, infection can manifest as toxoplasmic encephalitis and blindness. Additionally, there are several neurological and psychiatric associations with toxoplasmosis, including but not limited to epilepsy and bipolar disorder. This commentary reflects on recent work by Johnson and colleagues (H. J. Johnson, J. A. Kochanowsky, S. Chandrasekaran, C. A. Hunter, et al., mSphere 10:e00216-25, 2025, https://doi.org/10.1128/msphere.00216-25) investigating the difference in neuronal response to *T. gondii* compared to neurotropic West Nile and Zika viruses. They identify unique neurological and immune signatures associated with the different infections, as well as overlapping pathways enriched regardless of pathogen type. This study provides insights into host signaling pathways that can be manipulated for therapies against toxoplasmosis.

## COMMENTARY

The immunological status of the brain has confused clinicians and scientists alike for decades. Early experiments suggested the central nervous system (CNS) does not have an immune response. However, newer studies have challenged this notion on the basis that immune suppression can result in massively increased susceptibility to CNS infections, as well as the detection of recruited immune cells to the CNS upon infection. However, extended inflammatory and immune signaling can result in damage to self, and in the context of the brain, this can have devastating consequences in terms of function and survival. As such, the response must be finely tuned to strike a balance between being antimicrobial enough to clear a pathogen, while still affording protection from collateral damage. Additionally, although many CNS pathogens have a specific tropism for neurons, these cells have long been considered passive fighters in the response to infection—relying on exogenous immune effects in order to clear infection. For an organism like *Toxoplasma gondii* that can persist in the CNS of hosts for long periods of time, it is critical to understand the host immune response in more detail in order to identify ways to promote clearance of this parasite that is very good at evading the host immune response; additionally, this must be done with as little damage to the CNS as possible. Striking this balance requires an understanding of which CNS responses are generic to invading pathogens versus those that are specific to *T. gondii*. However, to date, there is still little clarity on the cell-autonomous immunity of neurons, particularly in response to varying pathogens.

Johnson et al. (1) have addressed this gap by comparing their own previously published data on neurons from brain sections of mice infected with *T. gondii* (2) and their new experiments on *T. gondii-*infected primary neuron cultures (PNCs), as well as a broader comparison of both these data sets against publicly available transcriptomic data sets from PNCs infected with two different neurotropic flaviviruses: West Nile and Zika viruses (3, 4). First, this allowed them to distinguish between neuronal and non-neuronal responses to *T. gondii*. This is critical because their previous work used laser capture microdissection to isolate neurons from tissue that had been injected with *T. gondii* proteins. This identified transcripts of CD8^+^ T cells and monocytes, indicative of contamination from these recruited surrounding cell types (2). The results from this comparison suggest that there are neuron-intrinsic immune mechanisms triggered upon *T. gondii* infection. These findings have been echoed by a study recently published by Chris Hunter’s group, where they used a neuron-specific STAT1 knockout mouse model to identify that neuronal STAT1 helps control parasite burden during chronic *T. gondii* infection in the brain (5). Additionally, further work from the Koshy lab has also demonstrated that neurons can clear parasites in response to IFNγ (6). Overall, these results confirm that neurons are intrinsically capable of mounting a cell-autonomous anti-parasitic response.

Secondly, the comparative analysis performed by Johnson et al. also allows identification of transcriptional changes induced in neurons specifically by *T. gondii* in comparison to viral CNS infections. A broad assessment of all data sets reveals an upregulated inflammatory signature (including type I and type II interferon signaling) induced upon infection by both virus and parasites, as expected. In particular, the authors highlight the enrichment of CXCL10 (typically secreted in response to IFNγ) across all data sets. Given that CXCL10 serves as a chemoattractant for recruiting immune cells to infected neurons, it is most likely crucial for control of infection regardless of pathogen, which further warrants validation of neuronal CXCL10 and its role in mediating the control and clearance of CNS pathogens. Delving further into this analysis, there are also key differences in the neuronal response to viruses versus parasites. Only the *T. gondii* data sets, but not the viral ones, show downregulation of neuronal function pathways. In particular, enrichment of this pathway in the PNC data set suggests that this phenotype is not mediated by recruited/resident immune cells but rather by the parasite itself—perhaps in order to establish a better intracellular environment for long-term survival. This opens up exciting avenues for further research investigating the manipulation of specific neuronal cell types by the parasite. Overall, this study provides a broader landscape of neuronal responses to infection, underscoring the importance of investigating the host response to pathogens in the brain.
